# Irrigation water quality shapes soil microbiomes: a 16 S rRNA-based biogeographic study in arid ecosystems

**DOI:** 10.1038/s41598-025-13705-w

**Published:** 2025-08-04

**Authors:** Mennatallah S. Abdelkader, Salah Abdalla, Ali A. Abdelrahman, Ibrahim A. Amin, Mohammed Ramadan, Mohammed Salah

**Affiliations:** 1 Department of Microbiology and Immunology, Faculty of Pharmacy, Port-Said University, Port-Said, Egypt; 2 Department of Microbiology and Immunology, Faculty of Pharmacy, Suez Canal University, Ismailia, Egypt; 3 Department of Microbiology and Immunology, Faculty of Pharmacy, Al-Azhar University, Assiut, Egypt

**Keywords:** Soil microbiome, Irrigation water quality, 16S rRNA sequencing, Microbial adaptation, Bioremediation, Salinity stress, Ecology, Environmental sciences

## Abstract

Soil microbiome plays a crucial role in ecosystem; however, the responses of the soil microbiome to nonconventional irrigation water sources remain poorly understood. This study employed 16 S rRNA sequencing to investigate microbial community shifts in soil samples collected from four geographically distinct locations affected by different irrigation water sources: saline ground water affected by seawater (SW), a brackish water lake (BW), a wastewater drain (WW), and a freshwater canal that receives inflows from multiple agricultural drains (FW). Our findings revealed distinct microbial signatures shaped by water quality, with Firmicutes dominating WW soils (49.2%) due to metal resistance (DESeq2, *p =* 3.67 × 10^− 4^), whereas *Chloroflexi* and *Cyanobacteria* thrived in BW environments (LEfSe, LDA > 4, *p =* 8.23 × 10^− 6^), reflecting adaptations to chloride-rich conditions. FW soils enriched Acidobacteria and Verrucomicrobia, which are associated with moderate salinity and nutrient cycling, whereas SW samples harbored halotolerant *Actinobacteria* and Deinococcus-Thermus (DESeq2, *p =* 1.47x^− 05^). Statistical analyses revealed key potential biomarkers, including *Streptococcus* (WW, DESeq2 *p =* 3.67x^− 24^), *RB41* (BW, LEfSe *p =* 1.62x^− 13^), and *Candidatus_Udaeobacter* (SW, DESeq2 *p =* 1.47x^− 05^). Physicochemical drivers such as salinity (R² =0.319, *p =* 0.00041) and heavy metals (Pb/Mn in WW) strongly influence community structure. Notably, WW irrigation reduced alpha diversity (Shannon index: 4.79–5.41 vs. 6.65–7.43 in FW; Kruskal-Wallis *p =* 0.0056), highlighting pollutant-induced stress. These findings highlight the balance between water reuse and soil health, offering a foundation for microbiome-driven bioremediation approaches in arid environments. By utilizing native, stress-resilient microbial communities, our research promotes sustainable agricultural practices in water-limited regions.

**Supplementary Information:**

The online version contains supplementary material available at 10.1038/s41598-025-13705-w.

## Introduction

Soil is a dynamic ecosystem that harbors diverse microbial communities, which often form microcolonies closely associated with soil particles. Factors such as micropore size distribution, the availability of essential microelements, and the presence of pollutants can significantly influence microbial activity, community composition, and overall ecosystem functioning^1^.

Climate change, tillage, and the excessive use of inorganic fertilizers and pesticides can disrupt natural selection, thereby altering soil bacterial abundance and diversity^2^. Several studies have revealed that irrigation water quality directly affects soil salinity and overall soil health^3^. Approximately 60% of the global population experiences water scarcity due to reduced rainfall and increasing water demand. To address water scarcity, many countries now rely on nonconventional water sources, such as treated wastewater, agricultural drainage, and saline groundwater, for irrigation^4^.

However, the impacts of these practices on soil microbial communities remain poorly understood, despite their potential to contribute to environmental pollution and affect crop productivity. Key contaminants include heavy metals from industrial activities, detergents from wastewater discharge, pesticides from agricultural practices, and sewage from domestic sources. The accumulation of these pollutants can pose significant environmental and health risks, affecting soil microbial communities and agricultural productivity^5^. Irrigation with treated wastewater can increase microbial metabolic activity despite potential chemical and pathogenic hazards^6^. However, other studies indicate that increasing the proportion of wastewater used for irrigation can increase antibiotic resistance gene (ARG) abundance in the rhizosphere, potentially altering microbial community structure and function^7^. Similarly, irrigation with saline water requires careful management, as salinity can shift the microbial community composition, often reducing microbial diversity and activity, thereby impacting soil fertility and crop health. Additionally, agricultural drainage water can accumulate salts, fertilizers, and pesticides as it moves through soils and drainage networks. Its reuse may necessitate treatment to remove these contaminants, which otherwise could negatively affect soil microbial communities and overall soil health^8^. Since only a small fraction of soil microbes can be cultured via conventional methods, bioinformatics approaches such as metabarcoding have become the most effective tools for studying soil environments. These methods enable the identification of uncultured microorganisms, providing deeper insights into microbial diversity, functional roles, interactions, and evolutionary relationships in various ecosystems^9^. Previous studies have primarily examined the effects of environmental conditions and irrigation water quality on plant and soil health^10–12^. However, there is a critical need to explore microbial interactions and their adaptive responses to extreme conditions and pollutants. This study investigated the effects of four commonly used nonconventional irrigation sources, saline groundwater, brackish water, untreated wastewater, and agricultural drainage-contaminated freshwater on soil microbial communities. By analyzing bacterial community shifts under these nonconventional irrigation regimes, we provide a comparative framework for understanding how soil microbiomes adapt to nonconventional water sources. Through detailed examination of microbial structure and selective adaptation patterns, we aimed to correlate the presence of specific taxa with distinct environmental stressors. This insight paves the way for targeted microbial community modulation as a bioremediation strategy, leveraging naturally occurring microbial resilience to counteract the effects of water scarcity, soil salinization, and other environmental challenges. Our approach is inspired by real-world adaptation processes, offering a foundation for sustainable solutions in water-limited agricultural systems.

## Methods

### Sample collection

Soil samples were collected in duplicate (two copies for each sample) from precisely georeferenced locations across Egypt between February and March 2022. The samples were categorized into four groups based on the type of irrigation water or proximity to the respective water source: SW, BW, WW, and FW. SW group: Soil samples were taken from areas near the seashore, influenced by saline groundwater in the Port Said Governorate 31° 16’ 40.5" N 32° 16’ 07.6" E, where the soil is affected by the Mediterranean Sea. BW group: Samples were collected from soil near water lagoons characterized by brackish water, specifically from fish farms near Manzala Lake, a brackish water lagoon 31° 13’ 57.7" N 32° 13’ 24.1" E and 31° 07’ 03.9" N 32° 12’ 04.3" E. WW group: This group included soil samples irrigated with wastewater from the Bahr El Baqar drain, which carries effluents from industrial, domestic, and agricultural activities 31° 01’ 01.5" N 32° 12’ 15.7" E and 31° 05’ 08.1" N 32° 12’ 04.0" E. FW group: Samples were obtained from soils irrigated with freshwater from the Ismailia Canal, which receives inflows from multiple agricultural drainage effluents 30° 34’ 03.2" N 32° 13’ 57.3" E. At each site, two soil samples were collected, one near the irrigation source and one approximately 100 m inland.

Coordinates were recorded via Google Earth Pro (version 2022; Maxar Technologies, US Navy NGA) to ensure spatial accuracy. The geographic map was created using the leaflet package (v2.1.2) for interactive visualization^13^. Base map data were sourced from OpenStreetMap (ODbL license) (Additional information: Table S1, Figure S1). The samples were promptly transported to the laboratory in ice-cooled containers to preserve microbial integrity. Upon arrival, each sample was split into two portions: one for physicochemical analysis and the other stored at − 80 °C for DNA extraction and PCR amplification.

### Physicochemical properties investigation

Physicochemical analyses were conducted at the Soil Research Laboratories, Faculty of Agriculture, Suez Canal University, according to previously described methods^14^. The analyses included measurements of the soil texture, pH, electrical conductivity (EC), sodium adsorption ratio (SAR), total organic carbon (TOC), organic matter (OM), total nitrogen (TN), total potassium (TK), total phosphate (TP), major cations (Na⁺, K⁺, Mg²⁺, Ca²⁺), anions (HCO₃⁻, Cl⁻, SO₄²⁻), and heavy metals (Cd, Co, Mn, Pb). The particle size distribution was assessed to classify the soil fractions into sand (2.0–0.05 mm), silt (0.05 –0.002 mm), and clay (< 0.002 mm), following the methods outlined in reference^15^ (Additional information: Table S2).

### DNA extraction, PCR amplification and 16 S rRNA gene sequencing

Microbial DNA was extracted via the DNeasy PowerSoil Kit (Qiagen, Cat. No. 12888, Valencia, USA) according to the manufacturer’s guidelines. The hypervariable V3-V4 regions of the 16 S rRNA gene were amplified via Illumina-adapted primers, as previously described^16–19^. These primers are as follows:

F < 5’TCGTCGGCAGCGTCAGATGTGTATAAGAGACAGCCTACGGGNGGCWG.

*R* < 5’GTCTCGTGGGCTCGGAGATGTGTATAAGAGACAGGACTACHVGGGTATCTAATCC’.

The quality and size of the PCR amplicons were verified via electrophoresis on a 1% agarose gel. thePCR amplicons were sequenced at IGA Technology Services (Udine, Italy) via the Illumina MiSeq platform (Illumina, USA).

### Processing and analysis of 16 S rRNA sequences

Initial bioinformatic processing included base calling, demultiplexing, and quality assessment. The raw sequencing data were processed via Bcl2Fastq v2.20 (Illumina) to convert the base call (BCL) files into FASTQ files and perform demultiplexing^20^. Adapter sequences were trimmed via Cutadapt v1.16^21^. Read quality was assessed via FastQC^22^.

Demultiplexed paired-end reads were imported into QIIME2 (version 2023.9) for downstream analysis^23^. Nonbiological sequences, including low-quality, marginal, and chimeric reads, were removed via the DADA2 plugin (Phred quality ≥ 25, max expected errors = 2), with truncation lengths set to 270 bp (forward) and 210 bp (reverse). Amplicon sequence variants (ASVs) were taxonomically assigned via a naïve Bayesian classifier trained on the SILVA reference database at 97% similarity^24^.

### Bacterial diversity analyses

QIIME2 scripts were applied to the filtered dataset to analyze microbial diversity, considering both intracommunity and intercommunity variations. Alpha diversity, reflecting within-sample microbial diversity, was assessed via the Observed and Shannon indices, each capturing distinct aspects of community heterogeneity. Statistical comparisons were performed via the Wilcoxon rank-sum test for pairwise group comparisons and the Kruskal-Wallis test for multiple-group analyses.

At the genus level, beta diversity, which represents between-sample variation in microbial community composition, was assessed via Principal Coordinate Analysis (PCoA) based on Bray-Curtis dissimilarity. The statistical significance of beta diversity differences was determined using PERMANOVA (Permutational Multivariate Analysis of Variance) for pairwise comparisons.

### Identification of differentially abundant genera and potential biomarkers

Differentially abundant genera were defined via genus-level data. Taxa were aggregated at the genus level via the tax_glom() function, and relative abundances were calculated. Differentially abundant taxa associated with irrigation water types were identified via DESeq2, applying an FDR-adjusted *p* threshold of ≤ 0.05. Potential biomarkers were defined by LEfSe (logarithmic LDA scores > 3.0 and α = 0.05)^25^. Receiver operating characteristic (ROC) curve analysis, performed via the pROC package, was used to quantify the discriminatory power of each genus (AUC values) to distinguish among irrigation water groups^26^. Furthermore, to explore the relationship between microbial community composition and environmental variables, canonical correspondence analysis (CCA) was performed via the vegan package. Before analysis, the abundance data were Hellinger-transformed to reduce the influence of dominant taxa^27^. The environmental variables were standardized to unit variance to ensure comparability. The significance of the CCA axes was evaluated via permutation tests (999 permutations, *p <* 0.05). Taxa with relative abundances less than 0.1% were excluded to minimize noise and enhance interpretability. Statistical significance was assessed via Wilcoxon rank-sum tests with false discovery rate (FDR) correction. Spearman correlation analysis (r ≥ ± 0.6, *p ≤* 0.05) was used to assess relationships among taxa. All analyses were performed in R (v4.3.0), utilizing phyloseq, tidyverse, ggplot2, vegan and pROC, with the results visualized via the MicrobiomeAnalystR package (version 2.0).

## Results

### Physicochemical properties of the collected samples

The soil moisture content was evaluated alongside texture, which varied across the sampling locations. The soil textures for each group were as follows: SW - silt, BW - sandy clay, WW - clay, and FW - sandy clay loam. Analysis revealed that the concentrations of cobalt (Co) and cadmium (Cd) were below the detection limits, whereas lead (Pb) was detected exclusively in the WW samples. The manganese (Mn) concentrations varied across the samples, with the highest levels (580–600 mg/kg) observed in the WW group. All the samples presented pH values ranging from 7.44 to 7.95, with the lowest value for BW and the highest for FW. In addition, the group BW samples presented the highest values for cations (Na^+^, K^+^, Mg^2+,^ and Ca^2+^) and Cl^−^ anions whereas the SO_4_ ^− 2^ anions presented the highest values in the SW group. TN, OC, OM, and TK, had the highest concentrations in the WW group followed by those in the group FW. Only the TP concentrations were very high in the FW group.

Soil salinity was assessed via SAR and EC, dS/m. The highest values were recorded in the SW group (SAR: 28.57; EC: 26 dS/m), followed by BW (20; 24 dS/m) and WW (14.84; 15.5 dS/m). In contrast, the FW soils presented the lowest salinity levels (SAR: 4.3; EC: 4.99 dS/m).

### Irrigation type of water significantly drives patterns of bacterial diversity

A total of 7,592,236 raw sequences were generated from 19 duplicate samples. Following quality filtering and chimera removal via the DADA2 plugin, 6,352,522 high-quality reads (83.67%) remained and were used for downstream analyses. Alpha-rarefaction analysis confirmed adequate sequencing depth across all the samples (Additional information: Figure S2).

**Fig. 1:**
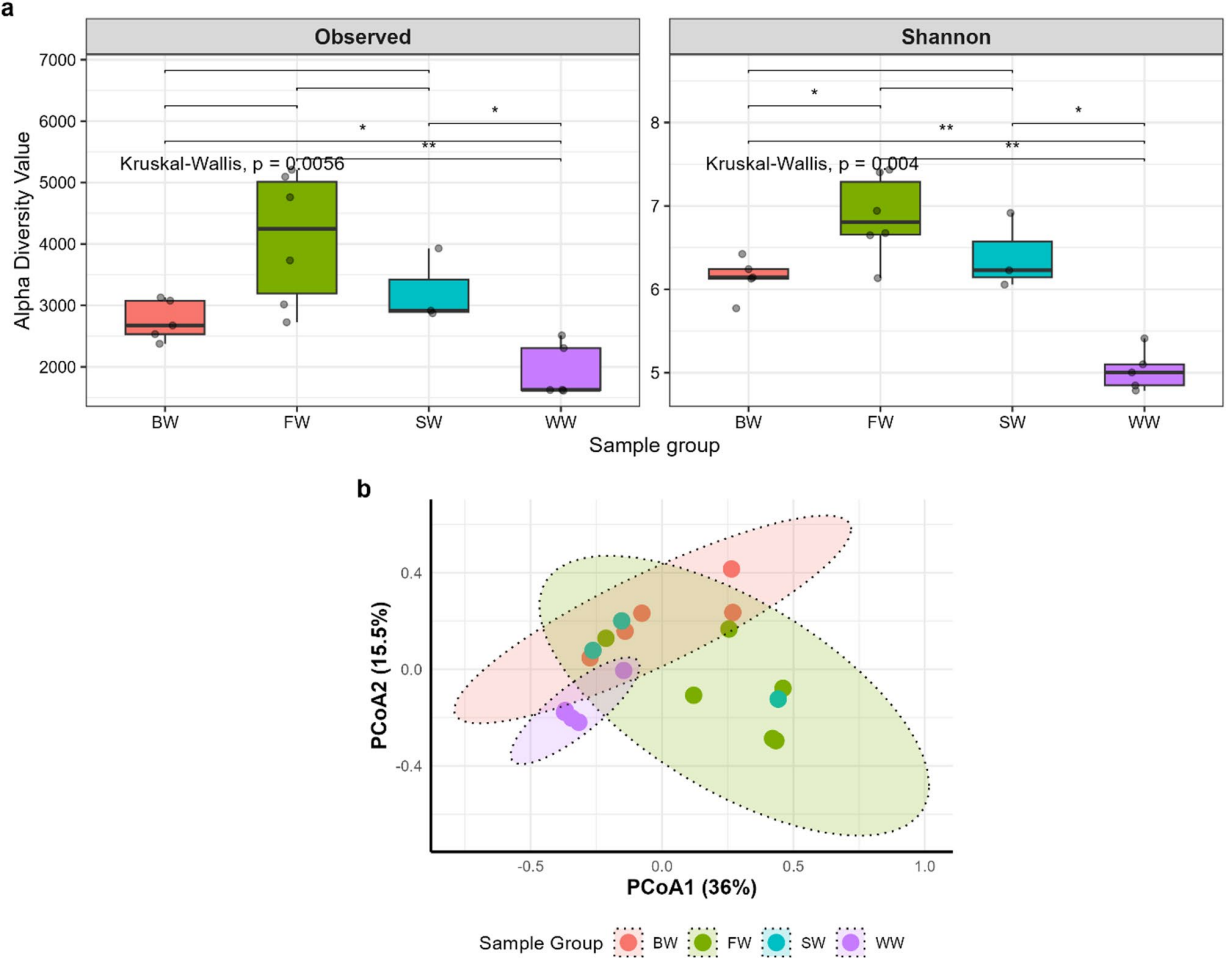
Bacterial diversity analyses of the microbial communities in the soils. (**a**) Boxplots defining the alpha diversity indices. The study groups (X-axis) are shown, and the Y-axis denotes the alpha diversity indices. The rhombus in each box indicates the median, the boxes define the interquartile range (IQR) between the 25th and 75th percentiles, and the whisker delimits the range. The pair-wise Wilcoxon rank-sum test was used to identify the statistical significance of pairwise comparisons and the Kruskal-Wallis test was used for group comparisons. Significant differences were indicated as either * (*p ≤* 0.05), or ** (*p ≤* 0.01). (**b**) PCoA plot for sample groups (PC1 1 = 36%; PC2 = 15.5%: PERMANOVA; F = 2.6906, R² = 0.34986, *p* = 0.002). The ellipses show significant clustering based on irrigation water type (*p* ≤ 0.01, PERMANOVA).

The relationships between the microbial community structure and irrigation water type were assessed via Observed and Shannon alpha diversity indices (Fig. 1).

The alpha diversity analysis revealed significant differences in the microbial community composition across the soil samples (Kruskal-Wallis test: Shannon, *p* = 0.004; Observed species, *p* = 0.0056) (Fig. 1a). Pairwise Wilcoxon tests with Benjamini-Hochberg correction revealed that compared with all the other groups, the WW group presented significantly lower diversity (Shannon: *p* < 0.01; observed species: *p* < 0.01), with the mean Shannon indices (4.79–5.41) markedly lower than those of the FW group (6.65–7.43; *p <* 0.05). BW displayed intermediate diversity (Shannon: 5.77–6.42) but was still distinct from WW (*p <* 0.05). Notably, SW showed no statistically significant differences from BW or FW (*p* > 0.05), despite numerical trends toward greater diversity (Shannon: 6.06–6.92. These results underscore that the irrigation water source (drainage vs. canal) and soil texture (clay vs. sand-clay-loam) are key drivers of microbial diversity. PERMANOVA at the genus level revealed a statistically significant difference in the microbial communities across the four soil groups. The model explained 35% of the variation in community composition (R² = 0.34986), with a *p* value of 0.002. **(**Fig. 1b**)**.

### Taxonomic profiling

**Fig. 2:**
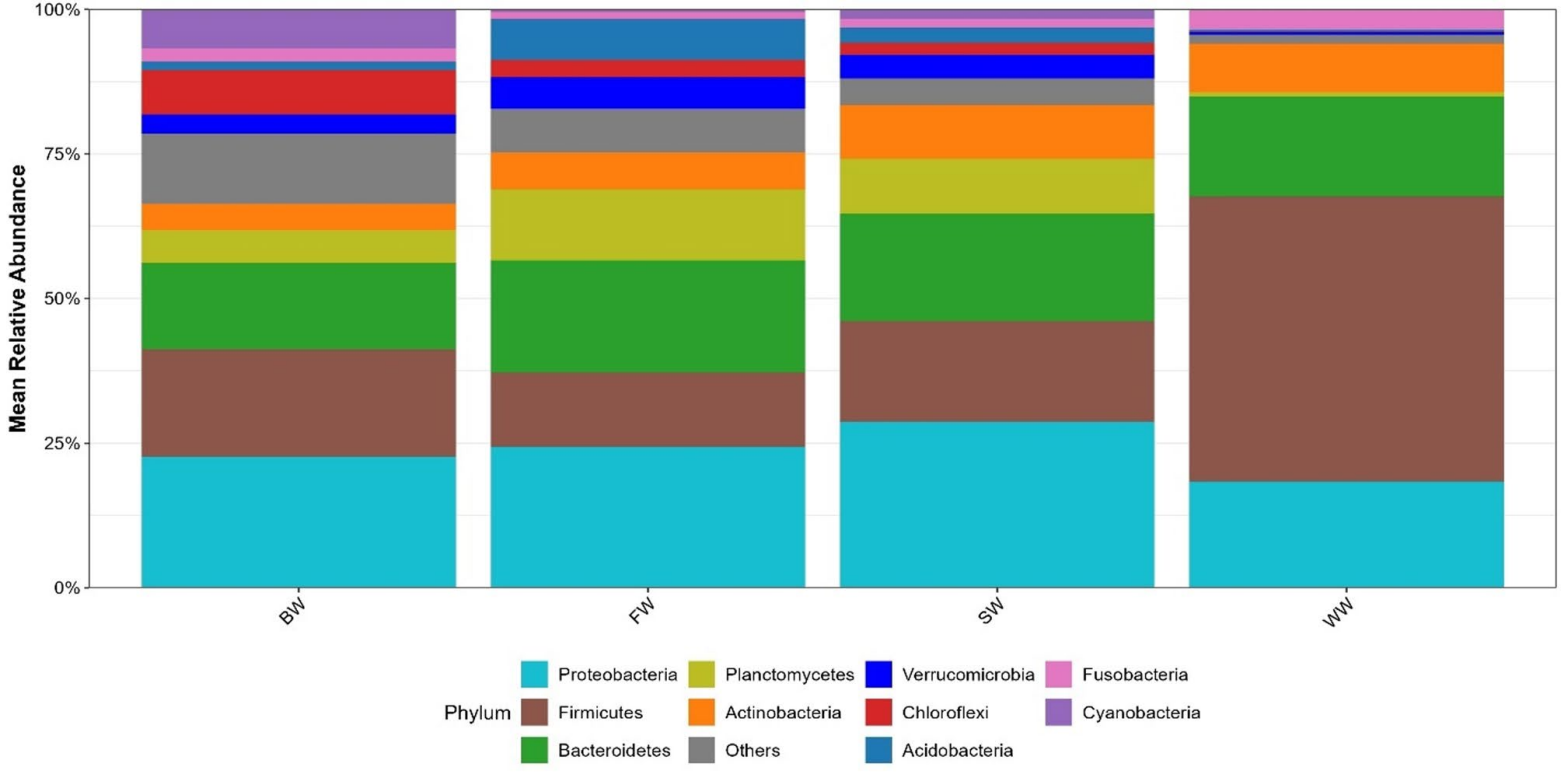
Effects of irrigation water type on the bacterial relative abundances of the main phyla in different soil samples. Four irrigation water sources were investigated: saline ground water affected by seawater (SW), a brackish water lake (BW), a wastewater drain (WW), and a freshwater canal that receives inflows from multiple agricultural drains (FW). The bar plot shows that the x-axis represents the relative abundance, and the y-axis represents the study groups.

Taxonomic classification of the 16 S rRNA reads identified 54 phyla, 131 classes, 323 orders, 563 families, and 1,401 genera. Phylum-level analysis revealed that Firmicutes, Proteobacteria, and Bacteroidetes were the dominant phyla across all the soil samples, although their relative abundances varied significantly by irrigation type (Fig. 2). Across all groups, Proteobacteria were consistently enriched compared with Bacteroidetes (Kruskal-Wallis test, *p =* 0.027). In the WW samples, Firmicutes were significantly enriched (49.21% ± 3.06%), exceeding the relative abundances of both Proteobacteria (18.32% ± 0.77%) and Bacteroidetes (17.41% ± 0.59%) (Wilcoxon rank-sum, *p =* 0.007). In contrast, Firmicutes were less abundant in the SW (17.54% ± 10.07%), BW (19.21% ± 14.5%), and FW (11.47% ± 7.52%) samples.

Distinct phylum-level signatures were associated with each irrigation group. The SW samples presented relatively high abundances of Actinobacteria, Calditrichaeota, and Deinococcus-Thermus. The BW samples were enriched in Chloroflexi, Cyanobacteria, and the archaeal phylum Cloacimonetes. The FW samples were characterized by elevated levels of Acidobacteria, Verrucomicrobia, and Planctomycetes. Conversely, WW samples presented reduced abundances of these same phyla, but increased levels of Fusobacteria and Firmicutes.

Some phyla exhibited a unique presence in specific groups. For example, BW samples uniquely harbored Cloacimonetes and Hadesarchaeaeota (archaea). The phylum *GAL15* was uniquely detected in FW samples (Additional information: Figure S3). No significant differences were detected between samples collected near versus away from irrigation sources (Kruskal-Wallis test, *p* > 0.05).

### Distinct compositional and structural patterns of bacterial communities are related to irrigation type

**Fig. 3:**
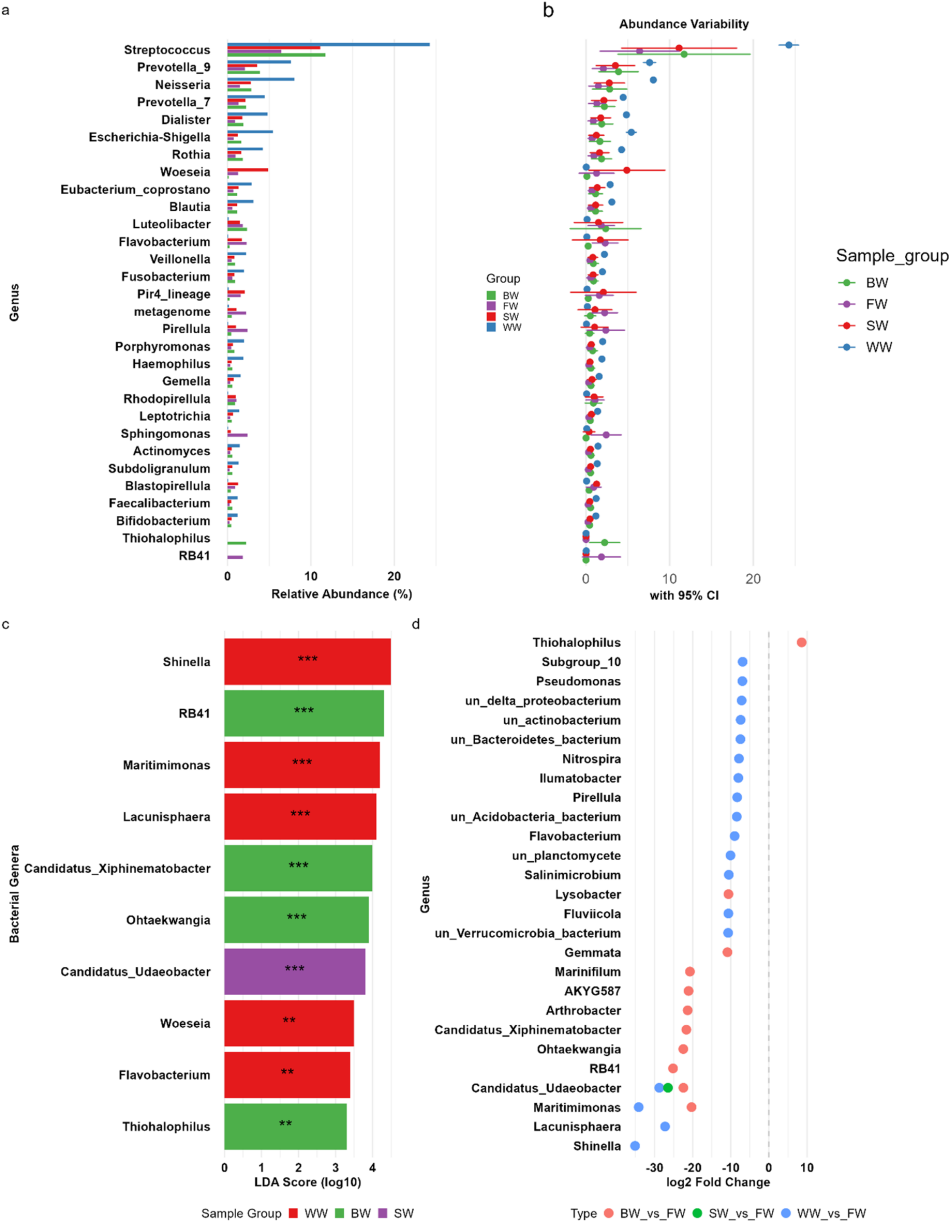
Differential abundance and biomarker analysis of soil bacterial genera across irrigation water types. Multi-panel visualization of bacterial genera that were differentially abundant across the four irrigation water sources. (**a**) Bar plot showing the mean relative abundance (%) of the top 30 genera across sample groups, with asterisks indicating statistical significance (*p* < 0.05, *p* < 0.01, *p* < 0.001). (**b**) Confidence interval plot displaying the variability (95% CI) in genus-level abundance across groups.(**c**) Volcano-style dot plot of the top 30 significantly differentially abundant genera based on DESeq2 analysis, showing log2 fold changes between groups. (**d**) LEfSe bar plot highlighting genera with significant LDA scores (log10) as potential biomarkers for each irrigation group, with significance levels annotated.

The abundance of the dominant genera varied significantly across the different sample types (WW, FW, BW, SW) (Fig. 3a). In WW, genera such as *Streptococcus*, *Prevotella_9*, and *Neisseria* were highly abundant. FW shows a different profile, with genera such as *Luteolibacter* and *Woeseia* being more prominent. The BW (brackish water) and SW (seawater) groups exhibit unique patterns, with *RB41* and *Thiohalophilus* being significant in BW, whereas Candidatus*_Udaeobacter* and *Maritimimonas* were notable in SW (Fig. 3b).

LEfSe analysis identified several genera as statistically significant biomarkers for each sample group (Fig. 3c). For example, *Shinella*, *Maritimimonas*, and *Lacunisphaera* were highly significant in the WW group, suggesting their potential role in wastewater ecosystems. The BW group featured *RB41*, *Candidatus_Xiphinematobacter*, and *Ohtaekwangia* as key biomarkers, highlighting their association with brackish environments. The SW group was significant for *Candidatus_Udaeobacter*, which may thrive under high-salinity conditions.

DESeq2 analysis of the microbial communities across different irrigation water groups (WW, FW, BW, SW) revealed significant differential abundances (*p <* 0.05) of key bacterial genera that reflect distinct environmental adaptations (Fig. 3d). In WW, nutrient-rich conditions favored significant enrichment of *Streptococcus* (*p =* 3.67e-24), *Prevotella_9* (*p =* 1.19e-16), and *Escherichia-Shigella* (*p <* 0.001), genera known for thriving in organic-rich environments. FW showed markedly different communities dominated by *Luteolibacter* (*p =* 0.0025) and *Woeseia* (*p =* 0.00069), which are characteristic of less saline, oligotrophic conditions. BW presented unique signatures including those of RB41 (*p =* 1.62x^− 13^) and *Thiohalophilus* (*p =* 0.00079), reflecting adaptations to moderate salinity. The SW group was distinguished by marine-associated taxa such as *Candidatus_Udaeobacter* (*p =* 1.47x-^05^) and *Woeseia* (*p =* 0.0022), demonstrating clear marine influences. Notably, there was a strong negative correlation (*p <* 0.001) between the salinity-sensitive freshwater taxa and the high-salinity groups. These patterns were further supported by LEfSe analysis, which identified additional group-specific biomarkers including *Lacunisphaera* (LDA > 4, *p =* 2.51x^− 16^) for WW and *Ohtaekwangia* (LDA > 4, *p =* 2.07x^− 13^) for BW.

### Microbial cooccurrence and environmental correlations

**Fig. 4:**
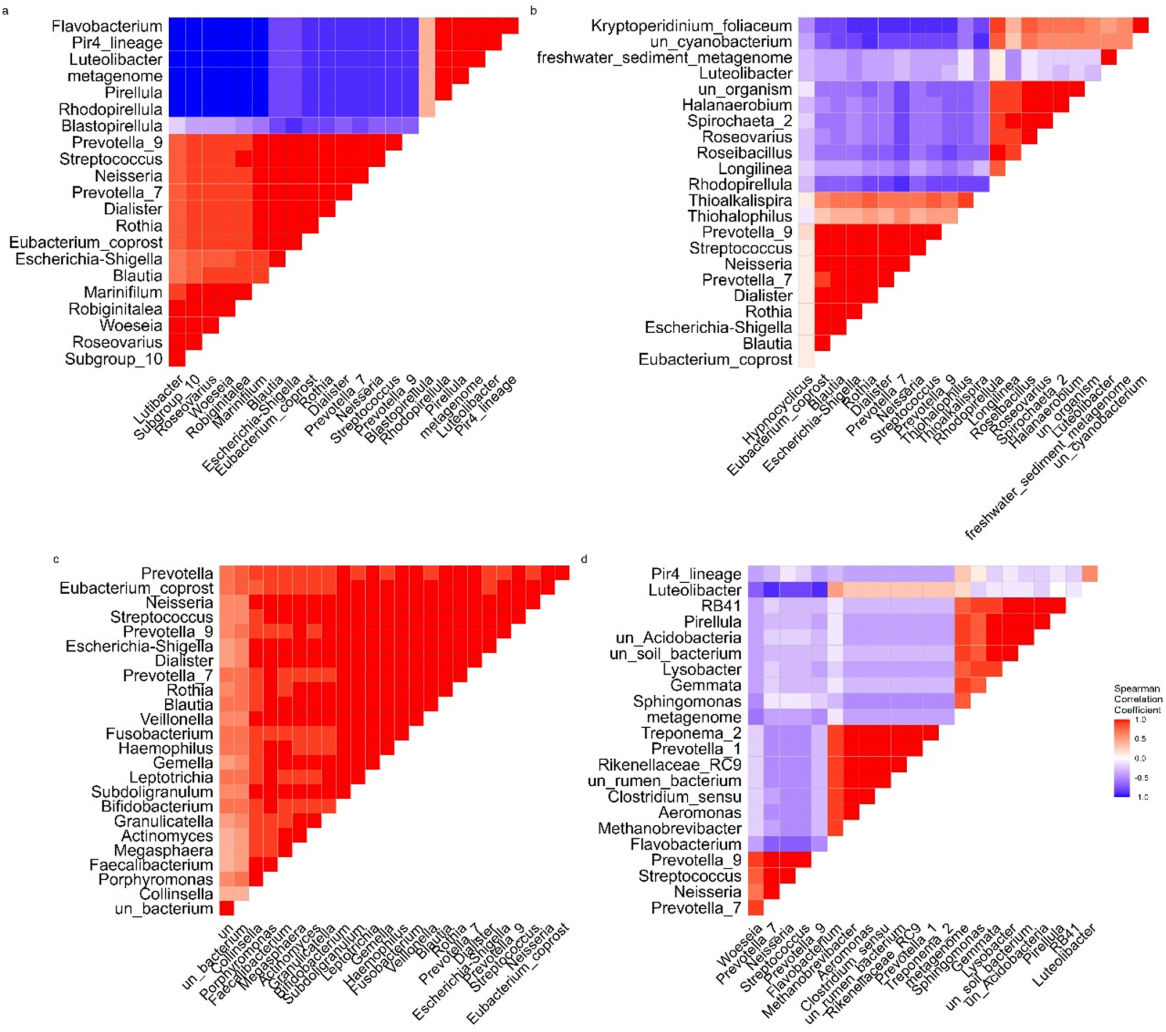
Hierarchical clustering of Spearman correlation matrices reveals distinct co-occurrence patterns across microbial and environmental datasets. Correlation heatmaps showing pairwise Spearman correlations between variables. (**a–d**) represent heatmaps for SW, BW, WW and FW, respectively. The variables were hierarchically clustered, and the correlations are displayed in the upper triangle (blue = -1, white = 0, red = + 1). The color gradient represents Spearman’s correlation coefficients (*r*) ranging from strong negative (blue) to strong positive (red) associations. All heatmaps share the same color scale for comparative analysis.

The correlation structure of bacterial taxa within distinct aquatic environments revealed how bacterial genera cooccur or interact within each environment. In SW (Fig. 4a), a clear cluster emerged among *Eubacterium_coprostanoligenes_group*, *Escherichia-Shigella*, and *Prevotella_9*. In contrast, taxa such as *Woeseia* and *Pirellula* appeared more isolated. BW reveals a different structure, with *Thiotrichales*, *Thioalkalispira*, and *Steroidobacter* forming a tight cluster (Fig. 4b). Another notable group included *Escherichia-Shigella* and *Eubacterium_coprostanoligenes_group*, suggesting some overlap with taxa found in other environments. WW shows a strong cluster of gut- and anaerobic-associated taxa such as *Rikenellaceae_RC9_gut_group*, *Prevotellaceae_UCG-001*, and *Methanobrevibacter*, which are typical of human-impacted or sludge-rich environments (Fig. 4c). Additional clusters such as un_Acidobacteria_bacterium_subgroup_10 and Clostridium_sensu_.

stricto_1 were detected in the WW group. Finally, FW displays a highly cohesive cluster involving *Eubacterium_coprostanoligenes_group*, *Prevotella_9*, *Escherichia-Shigella*, *Fusobacterium*, and *Streptococcus* (Fig. 4d).

**Fig. 5:**
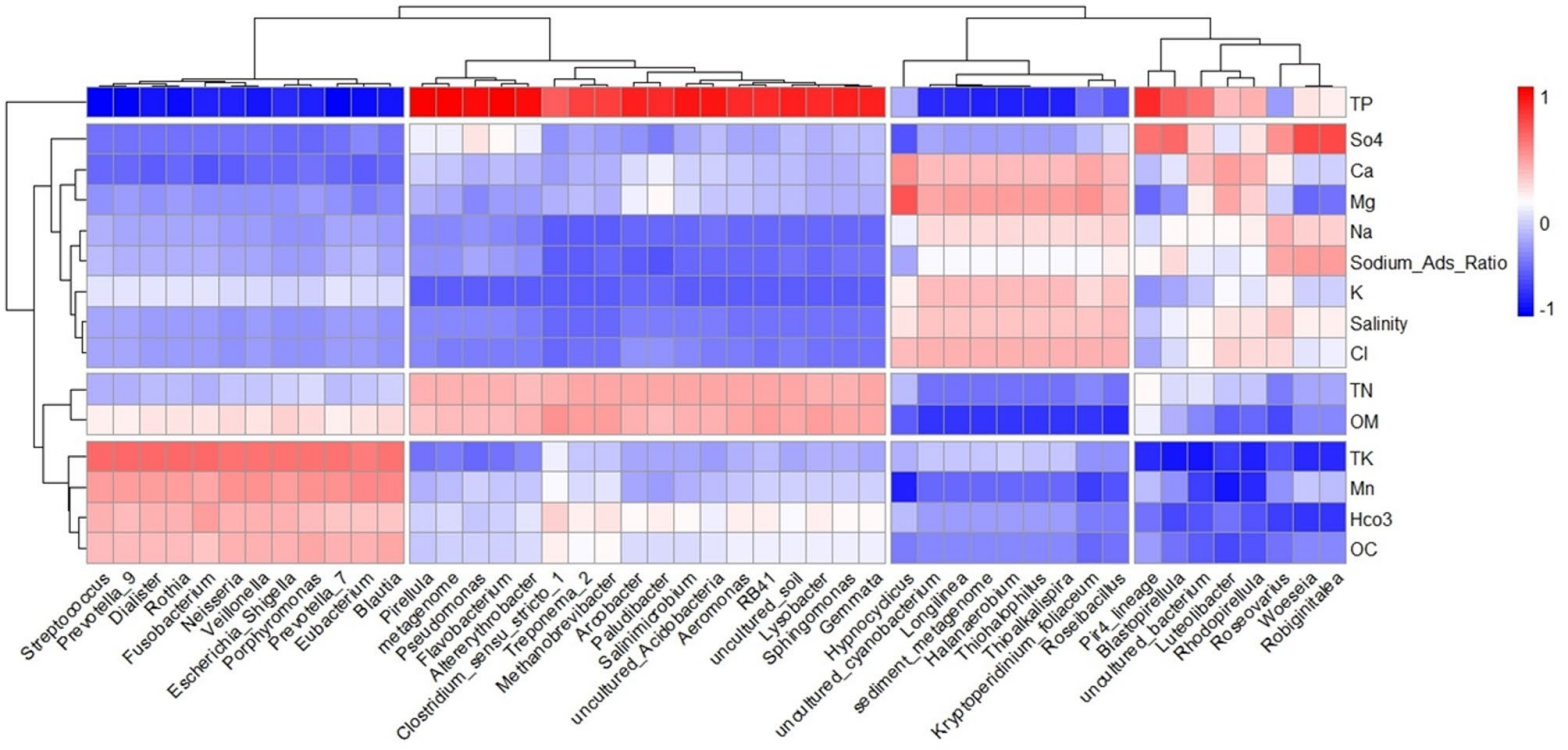
Correlation heatmap between the top 50 bacterial genera and the concentrations of the essential elements in soil samples. The heatmap scale is color coded in dark red for strong positive correlations and dark blue for negative correlations.

Further analysis of the relationships between physicochemical properties and bacterial genera revealed significant correlations (Fig. 5). Genera such as *Pirellula*, *Pseudomonas*, *Woeseia*, and *Altererythrobacter* were strongly positively associated with high concentrations of TP, TN, and OM. In contrast, *Woeseia* and *Robiginitalea* were positively linked to sulfate ions, whereas *Hypnocyclicus* correlated with magnesium but exhibited a negative relationship with manganese (Mn). Notably, certain genera, including *Hypnocyclicus*, *Cyanobacterium*, and *Longilinea*, were negatively correlated with OM, TN, TP, and bicarbonates, but demonstrated tolerance to high salinity conditions, including elevated levels of calcium, magnesium, sodium, potassium, and chloride ions.

Microbial biomarker analysis via AUC values revealed taxa specifically associated with distinct irrigation water sources, demonstrating the critical role of water quality in shaping soil microbial ecology (Additional information: Figure S4). High-salinity environments, BW and SW, were enriched with halophilic and marine-adapted genera: Group BW featured *Thiohalophilus* (AUC = 0.801; *p =* 0.048) and *Geoalkalibacter* (AUC = 1.00; *p =* 0.029) adapted to extreme salinity (24–25 dS/m) and sodium levels (140–150 mg/L), whereas SW contained marine specialists *Woeseia* (AUC = 0.958) and *Sediminicola* (AUC = 0.938) thriving under high salinity (19–20 dS/m) and ECs (28–29). In contrast, WW contained clinically significant genera including *Fusobacterium* and *Escherichia-Shigella* (AUC = 1.00; *p =* 0.016), indicating that the contamination risk was corroborated by the presence of Pb. FW maintained a balanced microbiome dominated by *Blastocatella* (AUC = 0.987) and *Massilia* (AUC = 0.923), reflecting favorable conditions with lower salinity (4–5 dS/m) and EC ratios.

### Environmental drivers of microbial community structure

**Fig. 6:**
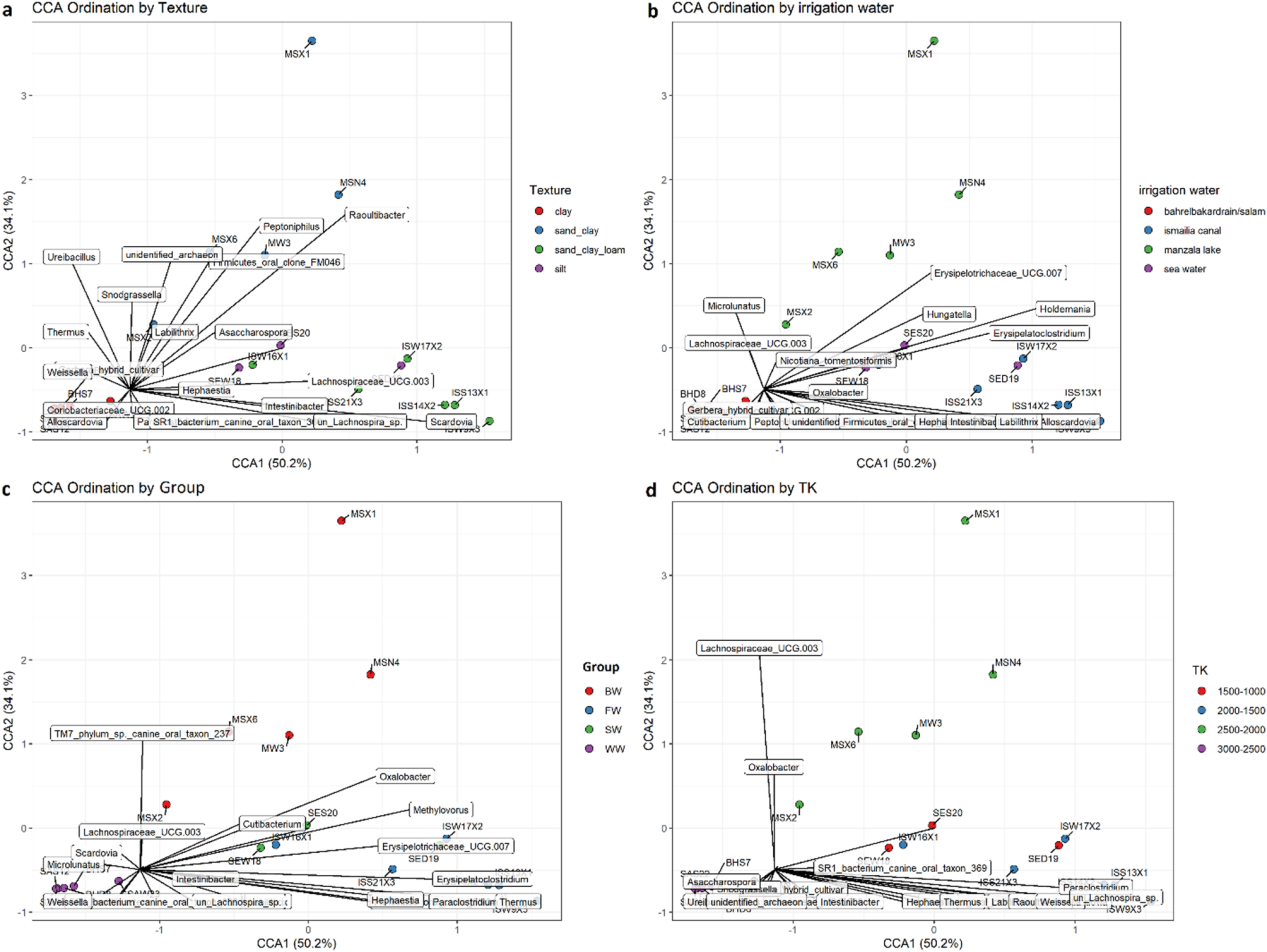
Canonical correspondence analysis (CCA) of microbial communities according to significant environmental variables. The ordination plots display the distribution of the microbial community samples along the first two canonical axes (CCA1 and CCA2), with arrows indicating genera contributing to community variation. The CCA ordination highlights significant environmental variables that are statistically associated with microbial composition (*p*_adj_ < 0.05). Texture, irrigation water, and sample type were highly significant (*p*_adj_ = 0.0073, F = 2.34), whereas TK was moderately significant (*p*_adj_ = 0.0165). **(a)** CCA by soil texture, **(b)** CCA by irrigation water source, **(c)** CCA by treatment group, **(d)** CCA by total potassium (TK).

Complementing these findings, CCA analysis demonstrated strong environmental filtering of the microbial communities (Fig. 6; Additional information: Figure S5). The key drivers included soil texture, irrigation water type, and salinity (R² = 0.319, *p =* 0.00041), which partitioned taxa into distinct ecological niches: *Ureibacillus* and *hybrid_cultivar* clustered in moderate-texture soils (WW: Ca = 45, Mg = 22.5, Na = 85 mg/L), whereas *Microlunatus* occupied saline/nutrient-limited zones. Archaeal taxa (*Unidentified_archaeon*) appeared peripherally, suggesting extremophilic lifestyles, and halotolerant genera (*Crinobacteriaceae_UCG.002*, *Holdēmania*) associated with high-solute samples (SW group: salinity = 2750, Cl⁻ = 1250 mg/L). *Snodgrassella* are linked to plant-rich microenvironments, whereas sensitive taxa (*Saccharospora*, *Nicotiana*-related groups) are excluded from high-salinity areas, which aligns with their low bicarbonate tolerance (HCO₃⁻: R² = 0.108, *p =* 0.622). These spatial patterns confirm texture and irrigation water as primary community architects while highlighting the niche specialization of stress-adapted (*Microlunatus*) versus organic-dependent (*Peptoniphilus*) taxa, providing a predictive framework for microbiome management in irrigated ecosystems.

## Discussion

Irrigation water plays a significant role in shaping the soil microbial community. The physical, chemical, and biological characteristics of the irrigation water influence the microbial composition, diversity, and functions in soil. Irrigation water can introduce new microbial taxa, including beneficial microorganisms, pathogens, or antibiotic-resistant bacteria, into the soil, depending on the water source. Industrial or wastewater irrigation may introduce heavy metals and pollutants, selectively promote metal-resistant microbes while suppress sensitive microbes. Some irrigation water sources, particularly wastewater or saline water, can alter soil salinity and pH, affecting microbial diversity and enzyme activities^28–32^. This study investigated the impact of nonconventional irrigation water sources on microbial soil communities in arid and semiarid regions. Our focus is on understanding microbial interactions and adaptive responses to extreme conditions and pollutants resulting from these irrigation practices.

First, the influence of different types of irrigation water on the physicochemical properties of the four groups of soil samples was investigated. The SAR and EC levels in all four groups exceeded the threshold defined by the US Salinity Laboratory, which considers normal soil to have an electrical conductivity (EC) of the saturation extract < 4dS m⁻¹ at 25 °C^33^. Consequently, we conclude that the use of nonconventional water resources increases SAR and EC levels, contributing to soil salinization. Furthermore, water scarcity alone exacerbates this issue by promoting salinity accumulation in the soil^34^.

Each irrigation type uniquely affected the soil properties. BW, enriched with industrial and domestic waste, introduces high levels of cations and chloride ions, leading to their accumulation in the respective soil group^35^. Similarly, saline groundwater (SW), the most saline irrigation source, contains elevated sulfate ions, further increasing soil salinity. This reduces soil bioavailability, poses toxicity risks to plants, and disrupts microbial balance^34,36,37^. Untreated wastewater, including sewage sludge, is known to contain high concentrations of heavy metals and OM^38,39^. Accordingly, irrigation with wastewater led to increased concentrations of manganese (Mn) and lead (Pb), along with elevated organic matter (OM), organic carbon (OC), total nitrogen (TN), and total potassium (TK) in the soil of the WW group^40^. However, when properly treated, wastewater can improve soil fertility and microbial activity by increasing bioavailability and supporting bioremediation^41^. The FW group presented the highest concentration of total phosphorus (TP), likely due to agricultural drainage containing phosphorus-rich herbicides, pesticides, and fertilizer industry effluents^42^. While this type of irrigation water may introduce resistant microbial phyla into the soil, it can also alter microbial community richness and composition, potentially reducing certain soil functions^43–45^.

Second, the physicochemical properties of irrigation water influence both the distribution and abundance of microbial phyla across soil types, thereby impacting alpha and beta diversity. The observed differences in alpha diversity highlight the substantial impact of irrigation water quality on soil microbial richness and evenness. The significantly lower diversity in WW irrigated soils may reflect environmental stressors such as elevated organic loads, heavy metals, or pathogen influx associated with untreated wastewater, all of which can suppress sensitive microbial taxa. This finding is consistent with previous studies that reported reduced microbial diversity in contaminated or heavily polluted soils^46,47^. Intermediate diversity in BW soils suggests a partial shift in microbial community structure due to moderate salinity and nutrient content^48^. Two possible explanations for this are as follows: (1) the microbial communities in brackish water lakes have biodegradation capabilities, allowing them to break down pollutants, and (2) the variations in salinity and water flow create distinct ecological niches, fostering diverse microbial communities adapted to fluctuating conditions^49^. The lack of significant differences between SW and both FW and BW, despite a trend toward greater diversity, may be due to adaptive microbial communities capable of tolerating saline environments, particularly in sandy or loamy soils.

Beta diversity analysis confirmed distinct clustering by irrigation type. The PERMANOVA results further confirmed that the irrigation water source plays a central role in shaping soil microbial communities^50^, explaining more than one-third of the total variation observed at the genus level. This implies that both water quality (e.g., chemical contaminants, nutrient load) and physical factors such as soil texture (clay vs. sand-clay-loam) jointly influence microbial assemblages^51^. Together, these findings reinforce the notion that sustainable water management is crucial not only for crop health but also for preserving soil microbial biodiversity^52^.

Next, we correlated the physicochemical properties of the irrigation water and soil with the taxonomic characteristics and relative microbial diversity via a metabarcoding approach. At the phylum level, three dominant bacterial phyla, Proteobacteria, Bacteroidetes, and Firmicutes, were identified as the core microbiome across all soil samples, which is consistent with previous studies^2,53^. However, Firmicutes exhibited an exceptionally high abundance (> 49%) in the WW samples, which can be explained by several hypotheses. Notably, *Firmicutes* exhibited an exceptionally high abundance (> 49%) in the WW samples, which can be explained by multiple factors. One reason is their ability to form endospores, enabling them to survive in highly polluted environments containing Mn and Pb^54^. Additionally, many Firmicutes species possess genes encoding hydrolytic enzymes such as cellulases, proteases, and phosphatases, which contribute to the degradation of organic matter and nutrient mineralization under anaerobic or metal-stressed conditions. Their metabolic versatility supports carbon turnover and phosphorus cycling in heavily polluted soils^55,56^. Another factor is the elevated OM as stated by Anderson et al.^57^, who reported a positive correlation between dissolved organic matter (DOM) and Firmicutes abundance in high-pH environments. In addition, the high concentrations of detected heavy metals may have suppressed Proteobacteria and Bacteroidetes, allowing Firmicutes to outcompete other phyla^58^. This hypothesis may also explain the reduced abundance of other phyla, including Acidobacteria, Verrucomicrobia, Planctomycetes, Chloroflexi, and Cyanobacteria which were negatively correlated with Firmicutes.

Across the four irrigation regimes, distinct environmental pressures led to unique microbial adaptations. SW soils, characterized by extreme salinity and elevated sulfate levels, favor extremophilic and stress-resistant taxa such as Actinobacteria, Deinococcus-Thermus and Calditrichaeota, which contribute to metal detoxification and oxidative stress mitigation in saline soils^59,60^. They also produce extracellular enzymes such as chitinases and ligninases, which facilitate organic matter decomposition^61^, making them important contributors to nutrient cycling and soil recovery in harsh, saline environments^62–66^. In contrast, BW soils, enriched with chloride and industrial waste, including pollutant-degrading Chloroflexi^67^, nitrogen-fixing Cyanobacteria^68^, sludge-associated Cloacimonetes^69^ and metabolically versatile archaea such as Euryarchaeota and Hadesarchaeota, which play key roles in carbon, nitrogen, and sulfur cycling^70,71^, presented the greatest microbial diversity. These taxa are capable of participating in biogeochemical cycles, including methane metabolism, toxic metal reduction, and nitrogen cycling, making them potentially valuable for bioremediation in contaminated and extremely anaerobic environments^72,73^. WW soils, which are heavily loaded with organic matter and toxic metals (Pb, Mn), exhibit reduced diversity and are dominated by Firmicutes, known for their endospore formation, anaerobic degradation capabilities, and resistance to heavy metals^74^. Moreover, FW soils, which are characterized by moderate salinity and high phosphorus levels, contain Acidobacteria, Planctomycetes and Verrucomicrobia, taxa involved in nutrient cycling, pH buffering, and plant-microbe interactions^42,70^. These phyla benefit from interactions with plants and other microbes^75^, and their presence suggests an adaptive response to alkaline and mildly saline conditions^76,77^. Notably, the FW group also presented a higher abundance of the poorly characterized phylum GAL15, which includes uncultivated microbes commonly found in moist, low-stress environments such as sediments, freshwater, and geothermal sites^78,79^.

These microbial profiles highlight how irrigation water quality can shape microbial community structure and function. These findings also suggest a potential bioremediation strategy to mitigate the effects of extreme salinity, radioactive contamination, and heavy metal pollution. For example, Chloroflexi^67^, Cyanobacteria^80^, Cloacimonetes^81^, and archaea such as Euryarchaeota and Hadesarchaeota could serve as promising candidates for treating chloride-rich or industrially contaminated soils. Likewise, Acidobacteria and Verrucomicrobia have potential for use in strategies aimed at restoring soils affected by salinization and alkalization^82^.

At the genus level, the composition of the dominant soil bacterial genera varied notably across the four irrigation sources, reflecting distinct environmental conditions. WW soils were enriched with *Streptococcus*, *Prevotella_9*, and *Neisseria*, genera commonly associated with fecal contamination and high organic matter and nutrient contents which is consistent with findings from wastewater-impacted environments^83,84^. These genera likely benefit from the elevated organic matter and pollutant load present in untreated wastewater and these genera along with *Escherichia-Shigella*, revealed significant differential abundances according to DESeq2 analysis. In contrast, FW, *Luteolibacter* (Verrucomicrobia) and *Woeseia* (Proteobacteria) were predominant likely due to the cleaner, less nutrient-rich conditions of freshwater sources. *Luteolibacter* has been documented as dominant in oligotrophic freshwater ecosystems and wetlands^85,86^. Similarly, *Woeseia*, initially isolated from coastal sediment, was also detected in aquatic habitats^87^. These genera reflect adaptation to lower nutrient inputs and are typically associated with less saline, oligotrophic conditions according to DESeq2, suggesting that FW supports a more balanced and less stress-tolerant microbial ecosystem. The BW microbiome, featuring *RB41* and *Thiohalophilus*, points to salinity-driven selection. *RB41* (Acidobacteria) is typically found in soils with fluctuations in salinity and moisture^88^. Although direct studies on *Thiohalophilus* are limited, its affiliation with sulfur metabolism under saline conditions is biologically consistent with that in brackish habitats^89^. These taxa are likely adapted to the intermediate salinity and fluctuating environmental parameters typical of the brackish water with which they are irrigated. Saline irrigation in the SW group promoted *Candidatus Udaeobacter copiosus* which is known as a highly efficient oligotroph that is dominant in carbon-poor terrestrial environments^90^, and *Maritimimonas*, a marine-derived genus that is found in the gut microflora of sea snails^91^, supports adaptation to high osmotic stress, reinforcing the strong selective effect of seawater irrigation^92^. Overall, irrigation type not only modulates the pathogen load (e.g., *Streptococcus* in WW) but also shifts the functional potential toward nutrient cycling (via sulfur/organic matter transformations) in BW and SW. These patterns highlight the necessity of tailoring irrigation systems to soil microbiome objectives to minimize pathogen transfer while supporting beneficial microbe mediated functions.

In addition to abundance and DESeq2 analysis, LEfSe analysis reinforces the ecological partitioning of microbial communities in response to distinct hydrological and chemical regimes, with implications for soil nutrient cycling, resilience, and microbial risk potential in reused water systems. These findings underscore the distinct microbial communities adapted to specific environmental niches, with many genera showing strong group-specific associations. LEfSe analysis identified these patterns by identifying group-specific biomarkers, such as *Shinella*,* Maritimimonas*^91^, and *Lacunisphaera*^93^ in WW, *Candidatus_Xiphinematobacter*,* RB41* and *Ohtaekwangia* in BW^94^, and *Candidatus_Udaeobacter* in SW. These genera may serve as indicators of water quality and ecosystem function^95,96^.

The hierarchical clustering of Spearman correlation matrices revealed distinct co-occurrence patterns of bacterial genera across different irrigation water types, reflecting the influence of environmental conditions on microbial interactions and community assembly. For example, in saline water (SW), a cluster involving E*ubacterium_coprostanoligenes_group*,* Escherichia-Shigella*, and *Prevotella_9* suggests that these taxa may share similar ecological niches or engage in mutualistic relationships within marine-influenced soils^97^. The presence of more isolated genera such as *Woeseia* and *Pirellula* indicates niche specialization or limited interactions with dominant taxa, highlighting the functional diversity in saline environments^98,99^. BW soils presented a unique cluster of *Thiotrichales*,* Thioalkalispira*, and *Steroidobacter*, suggesting adaptation to moderate salinity and possibly sulfur cycling or other chemolithotrophic processes common in transitional environments. Interestingly, genera such as *Escherichia-Shigella* and *Eubacterium_coprostanoligenes_group* appeared across multiple environments, indicating their ecological flexibility. In wastewater-irrigated soils (WW), clustering of *Rikenellaceae_RC9_gut_group*,* Prevotellaceae_UCG-001*,* and Methanobrevibacter* underscores the influence of human and fecal contamination, as these genera are often linked to the gut environment and anaerobic metabolism. The presence of additional clusters such as *Clostridium_sensu_stricto_1* and unclassified Acidobacteria reflects the complex, organic-rich, and possibly oxygen-limited conditions characteristic of wastewater-impacted soils^100^. The FW group environments revealed a highly integrated network, with co-occurrence of *Eubacterium_coprostanoligenes_group*, *Prevotella_9*,* Escherichia-Shigella*, *Fusobacterium*, and Streptococcus. This cohesive clustering may indicate synergistic interactions in oligotrophic or mildly nutrient-enriched conditions where microbial cooperation enhances nutrient processing and resilience^101^. Together, these patterns emphasize that microbial interaction networks are not random, but are structured by environmental pressures such as salinity, nutrient load, and organic pollution. Understanding these co-occurrence dynamics can help predict microbial community stability, resilience, and potential ecosystem functions under various irrigation practices.

Correlation analyses revealed how the soil elemental composition selectively nourishes specific microbial taxa. For example, *Woeseia* and *Robiginitalea* are thriving in sulfate-rich conditions because of their specialized metabolic capabilities. Conversely, oligotrophic and anaerobic genera such as *Hypnocydicus*, *Cyanobacterium*, *Longilinea*, *Halanaerobium*, *Thiohalophilus*, *Thioalkalispira*, *Kryptoperidinium foliaceum*, and *Roseibacillus* were negatively correlated with high OM, TN, TPh, TK, OC, Mn, and bicarbonate levels^82,102,103^.

AUC-based biomarker analysis identified taxa indicative of specific irrigation regimes: high-salinity environments (BW and SW) were enriched in halophiles (*Thiohalophilus*, *Geoalkalibacter*, *Woeseia*, *Sediminicola*) involved in sulfur oxidation and anaerobic respiration in sediments^104,105^. Conversely, WW harbored clinically relevant genera (*Fusobacterium*, *Escherichia-Shigella*), indicating fecal contamination and health risks. Freshwater systems (Ismailia canal/Group FW) feature beneficial taxa (*Blastocatella*, *Massilia*) that support organic decomposition and plant growth^106^. This highlights irrigation water’s dual role as microbial inoculant and environmental filter^107^.

Collectively, the CCA results demonstrated that salinity, soil texture, and irrigation type collectively governed the microbial community structure. The taxon distribution revealed niche specialization: *Microlunatus* and *Crinobacteriaceae* occupied saline/nutrient-limited zones consistent with polyphosphate accumulation and halotolerance^108^; archaea (*Unidentified_archaeon*) appeared in anaerobic/extremophilic niches; plant-associated *Snodgrassella* clustered with organic-rich soils; and stress-sensitive genera (*Saccharospora*, *Nicotiana*-related) were excluded from high-salinity regions^109,110^. These spatial patterns provide a predictive framework for microbiome management in irrigated agriculture.

Finally, these findings highlight how specific microbial taxa respond to distinct irrigation-induced stressors, offering a foundation for microbiome-informed soil management. For instance, the dominance of Firmicutes in WW-irrigated soils reflects their resilience to heavy metals and organic pollutants, suggesting their utility in the bioremediation of contaminated environments^111^. Similarly, the enrichment of Chloroflexi, Cyanobacteria, and archaea such as Euryarchaeota and Hadesarchaeota in brackish water soils indicates their potential roles in pollutant degradation, nitrogen cycling, and metal reduction^112,113^. In freshwater-irrigated soils affected by agricultural drainage, the presence of Acidobacteria and Verrucomicrobia, known for their roles in nutrient cycling and pH buffering, suggests their application in reversing soil salinization and alkalization^114,115^. These results pave the way for designing targeted microbial interventions aimed at revitalizing soil health, boosting nutrient cycling, and increasing crop tolerance in degraded and water-limited environments.

While this study provides valuable insights into irrigation-driven microbial shifts, several limitations should be acknowledged. The use of 16 S rRNA sequencing restricts functional resolution, limiting our understanding of microbial metabolic capabilities. Additionally, the study’s single-season sampling and geographic specificity constrain the temporal and spatial generalizability of the findings. Future research should incorporate multiomics approaches to unravel functional pathways and conduct longitudinal sampling to capture seasonal dynamics. Controlled experiments are needed to establish causal links between water quality and microbial responses. Expanding taxonomic coverage to include fungi and protists would offer a more comprehensive view of the soil microbiome. Furthermore, field trials testing bioaugmentation with stress-adapted taxa, such as Chloroflexi, in brackish soils, could validate their bioremediation potential. Conducting comparative analyses across arid regions worldwide would broaden the relevance of these results and contribute to advancing microbiome-informed strategies for sustainable agriculture in water-scarce environments.

## Conclusions

This study demonstrated that irrigation with nonconventional water sources significantly altered soil microbial communities, with each water type selecting for distinct microbial taxa adapted to specific environmental stressors. Saline groundwater favored halotolerant Actinobacteria and Deinococcus-Thermus, whereas brackish water enriched pollutant-degrading Chloroflexi, Cyanobacteria, and archaea such as Cloacimonetes. Wastewater irrigation led to the dominance of Firmicutes due to metal resistance, whereas freshwater with agricultural drainage selected for Acidobacteria and Verrucomicrobia adapted to moderate salinity. These microbial signatures are strongly correlated with soil physicochemical properties, particularly salinity, heavy metals, and nutrient levels. These findings highlight the potential of native stress-adapted microbes for bioremediation strategies in water-scarce regions. By leveraging these natural microbial adaptations, targeted approaches can be developed to mitigate soil degradation, enhance nutrient cycling, and maintain agricultural productivity under challenging irrigation conditions. This work provides a foundation for microbiome-based solutions to address the growing challenges of water scarcity and soil health in arid ecosystems.

## Supplementary Information

Below is the link to the electronic supplementary material.

Supplementary Material 1

## Data Availability

All used R scripts are available at https://gist.github.com/Mohammedramadan2012. The workflow used to train the RDP’s naïve Bayesian classifier is available at: https://github.com/mikerobeson/make_SILVA_db. Raw 16 S rRNA sequencing data have been deposited in the NCBI Sequence Read Archive (SRA) under accession number PRJNA828614. Data can be accessed at: https://www.ncbi.nlm.nih.gov/bioproject/?term=PRJNA828614.

## Abbreviations

ARG: Antibiotic resistance gene
ASVs: Amplicon sequence variants
AUC: Area under the curve
BW: Brackish lake water
CCA: Canonical correspondence analysis
Cd: Cadmium
Cl⁻: Chloride ion
Co: Cobalt
DOM: Dissolved organic matter
EC: Electrical conductivity
FDR: False discovery rate
FW: Freshwater contaminated with agricultural drainage
HCO₃⁻: Bicarbonate ion
Mn: Manganese
OC: Organic carbon
OM: Organic matter
Pb: Lead
PCoA: Principal coordinate analysis
PERMANOVA: Permutational multivariate analysis of variance
RDP: Ribosomal database project
ROC: Receiver operating characteristic
SAR: Sodium adsorption ratio
SO₄^2^⁻: Sulfate ion
SW: Saline groundwater
TOC: Total organic carbon
TN: Total nitrogen
TK: Total potassium
TP: Total phosphate
TPh: Total phosphorus
WW: Untreated wastewater
