# Supplementary material for: Irrigation water quality shapes soil microbiomes: a 16 S rRNA-based biogeographic study in arid ecosystems
Source: Sci Rep. 2025 Aug 4;15:28460. doi: 10.1038/s41598-025-13705-w (PMC12322162; doi:10.1038/s41598-025-13705-w)
Supplement: Supplementary file 1 — Supplementary Material 1 [file 41598_2025_13705_MOESM1_ESM.docx]

**Irrigation Water Quality Shapes Soil Microbiomes: A 16S rRNA-Based Biogeographic Study in Arid Ecosystems**

Mennatallah S. Abdelkader^1*^, Salah Abdalla^2^, Ali A. Abdelrahman^2^, Ibrahim A. Amin^3^, Mohammed Ramadan^3*^, Mohammed Salah^1^

^1^ Department of Microbiology and Immunology, Faculty of Pharmacy, Port-Said University, Port-Said, Egypt.

^2^ Department of Microbiology and Immunology, Faculty of Pharmacy, Suez Canal University, Ismailia, Egypt.

^3^ Department of Microbiology and Immunology, Faculty of Pharmacy, Al-Azhar University, Assiut, Egypt.

**Table S1: A summary of sampling locations and types of irrigation water source**

| **Location (no. of samples)** | **Sample group** | **Longitude** | **Irrigation water source** |
| --- | --- | --- | --- |
| **Port-Said (6)** | SW | 31°16'40.5"N  32°16'07.6"E | Sea water |
| **El Manzala (8)** | BW | 31°13'57.7"N  32°13'24.1"E | El Manzala Lake |
| **Biological Wastewater treatment station WWTS (2)** | BW | 31°07'03.9"N  32°12'04.3"E | EL Manzala Lake |
| **Bahr El Baqar (4)** | WW | 31°01'01.5"N  32°12'15.7"E | Bahr El Baqar drain/ El- Salam Canal |
| **El Husseiniya (6)** | WW | 31°05'08.1"N  32°12'04.0"E | Bahr El Baqar drain/ El- Salam Canal |
| **Ismailia/Field (12)** | FW | 30°34'03.2"N  32°13'57.3"E | Ismailia Canal |

**Table S2: Physicochemical characteristics of irrigation water samples collected from six locations in Egypt, representing four distinct water sources**

|  | irrigation water | pH | Electrical conductivity (EC) Salinity dS/m --ppm | SAR (sodium adsorption ratio | Na+ meq/l | K+ meq/l | Mg2+ meq/l | Ca2+ meq/l | HCO- meq/l | Cl- meq/l | SO42- meq/l | Pb (ppm) | Mn (ppm) | TN (ppm) | TP (ppm) | TK (ppm) | OC (ppm) | OM (ppm) | Sand % | Loam % | Clay % | Texture |
| --- | --- | --- | --- | --- | --- | --- | --- | --- | --- | --- | --- | --- | --- | --- | --- | --- | --- | --- | --- | --- | --- | --- |
| Port-Said | Sea water | 7.82 | 26/ 16870 | 28.57454907 | 145 | 1.3 | 16.5 | 35 | 5.5 | 138 | 55 | _ | 118.5 | 2860 | 238 | 1000 | 0.146 | 0.268 | 3 | 90 | 7 | silt |
| Al-Manzala | Manzala Lake | 7.47 | 24.3 / 15552 | 20.71428571 | 145 | 2 | 40 | 58 | 10 | 213 | 22 | _ | 84.81 | 3220 | 73.79 | 2000 | 0.562 | 0.969 | 48.4 | 8 | 43.6 | sand clay |
| Bahr Al-Bakar (Biological Waste water treatment station WWTS) | Manzala Lake | 7.45 | 24.6/ 15744 | 21.10796986 | 147 | 1.9 | 41 | 56 | 11 | 214 | 20 | _ | 84.87 | 3196 | 73.6 | 1998 | 0.57 | 0.968 | 49 | 7.5 | 43.5 | sand clay |
| Bahr Al-Bakar village | Bahr Al Bakar drain/Al-Salam Canal | 7.8 | 15.5 / 9920 | 14.84786235 | 82 | 1.1 | 21 | 40 | 11 | 120 | 20 | 5.302 | 580 | 5682 | 160 | 2520 | 1.695 | 2.9 | 26 | 17 | 57 | clay |
| Al Husseiniya village | Bahr Al Bakar drain/Al-Salam Canal | 7.95 | 8.07 / 5165 | 12.96158427 | 50.2 | 0.8 | 11 | 19 | 14 | 59 | 8 | _ | 153.1 | 2800 | 269.1 | 2600 | 0.843 | 1.453 | 34.7 | 13.8 | 51.5 | clay |
| Ismailia/Field | Al Ismailia Canal | 7.95 | 4.99 /3193 | 4.318004318 | 17 | 0.5 | 10 | 21 | 10 | 32 | 6.2 | _ | 93.67 | 3500 | 704 | 1750 | 0.911 | 1.588 | 65 | 11 | 24 | sand clay loam |

**Figure S1 Geospatial Distribution of Irrigation Water Sources in Northern Egypt.**


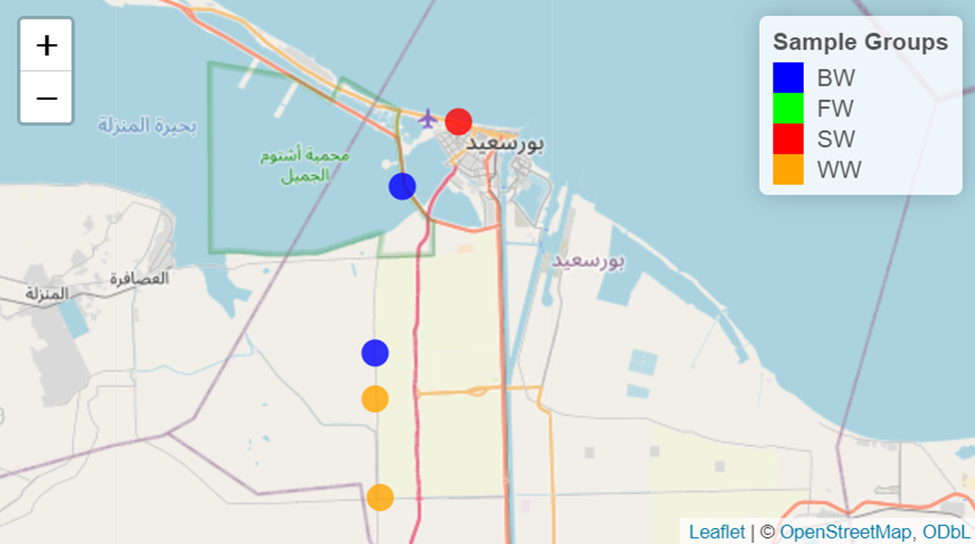


- **Figure S1:** Map showing the geographic locations of four distinct irrigation water sources in Northern Egypt. **SW:** Seawater-influenced soils (Port Said Governorate). **BW:** Brackish water (Manzala Lake). **WW:** Wastewater (Bahr El Baqar drain). **FW:** Freshwater (Ismailia Canal). Map generated using the leaflet package and OpenStreetMap base layers (ODbL license).

**Figure S2: Rarefaction Curves of Soil Microbial Communities Across Irrigation Water Types and Soil Textures**


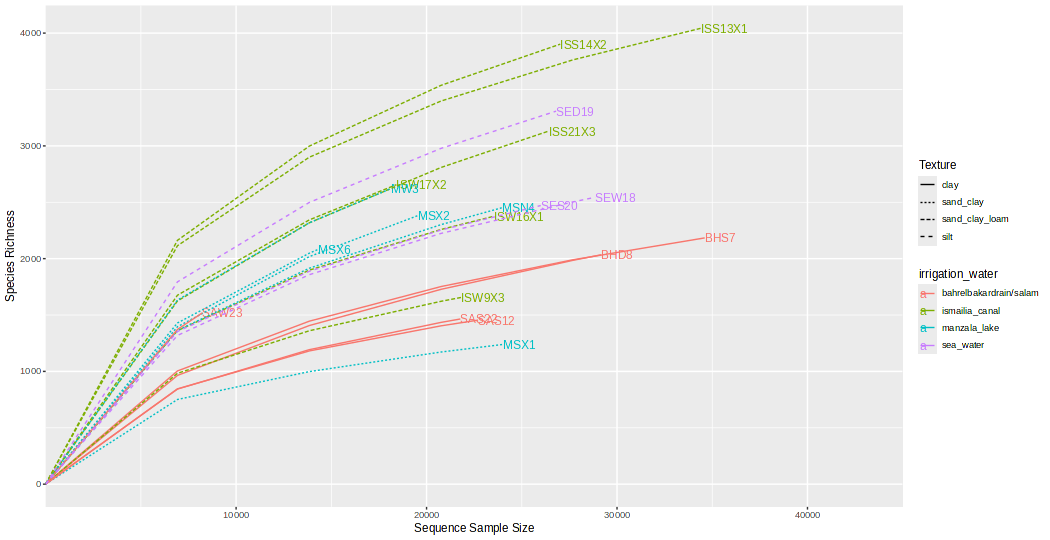


**Figure S2:** Rarefaction curves illustrate the relationship between species richness and sequencing depth across different combinations of soil textures (clay, sand clay, sand clay loam, silt) and irrigation water sources (Bahr El-Baqar drains/Salam Canal, Ismailia Canal, Manzala Lake, and seawater). Each curve represents a unique sample, with line styles denoting soil texture and colors indicating irrigation water type. The curves demonstrate microbial diversity saturation trends and highlight differences in richness among environmental conditions.

**Figure S3: Core Microbiome Composition Across Experimental Groups at Phylum and Genus Levels**


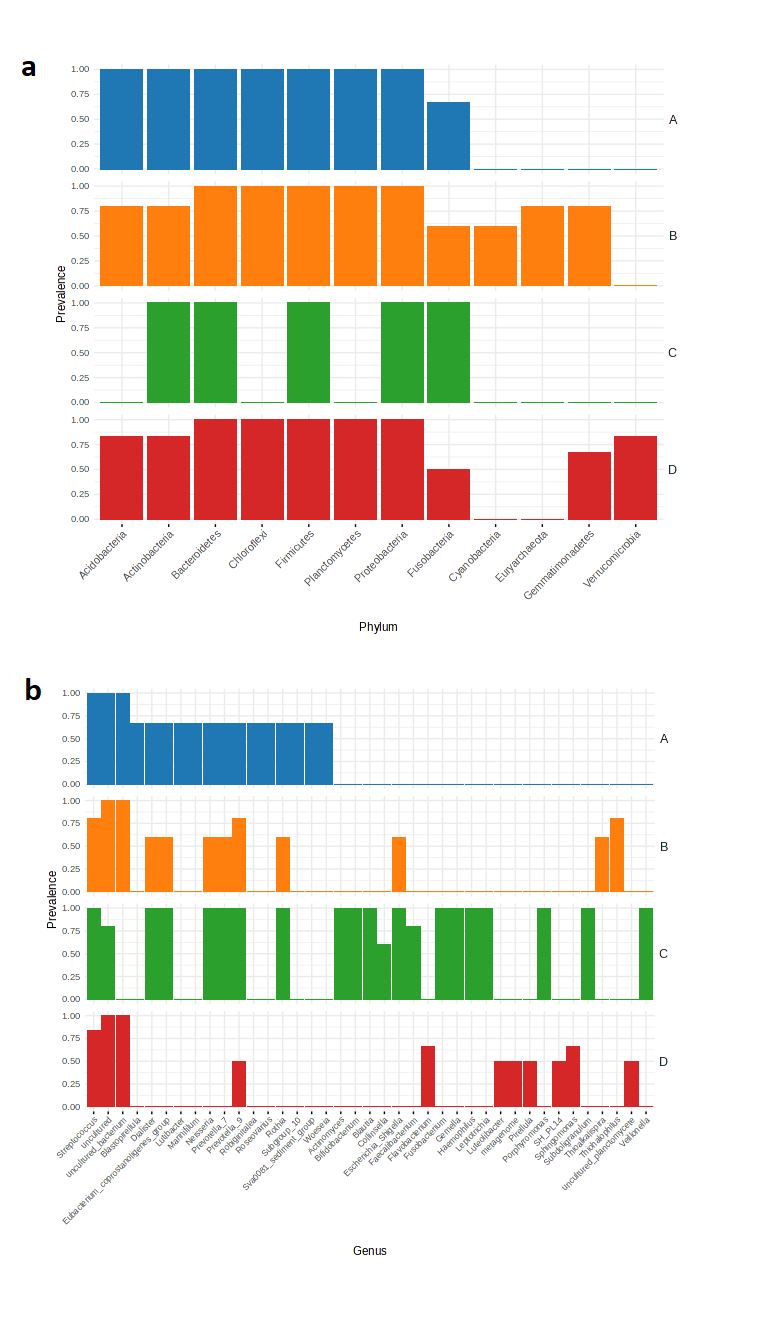


**Figure S3:** Bar charts represent the relative abundance of microbial taxa constituting the core microbiome across four experimental groups (A–D).

Panel (a) displays the distribution at the phylum level, revealing broad taxonomic trends, while

Panel (b) provides a more detailed view at the genus level, highlighting specific microbial signatures within each group.

**Figure S4: Top Discriminative Genera by Group: ROC Curve Analysis for Groups.**


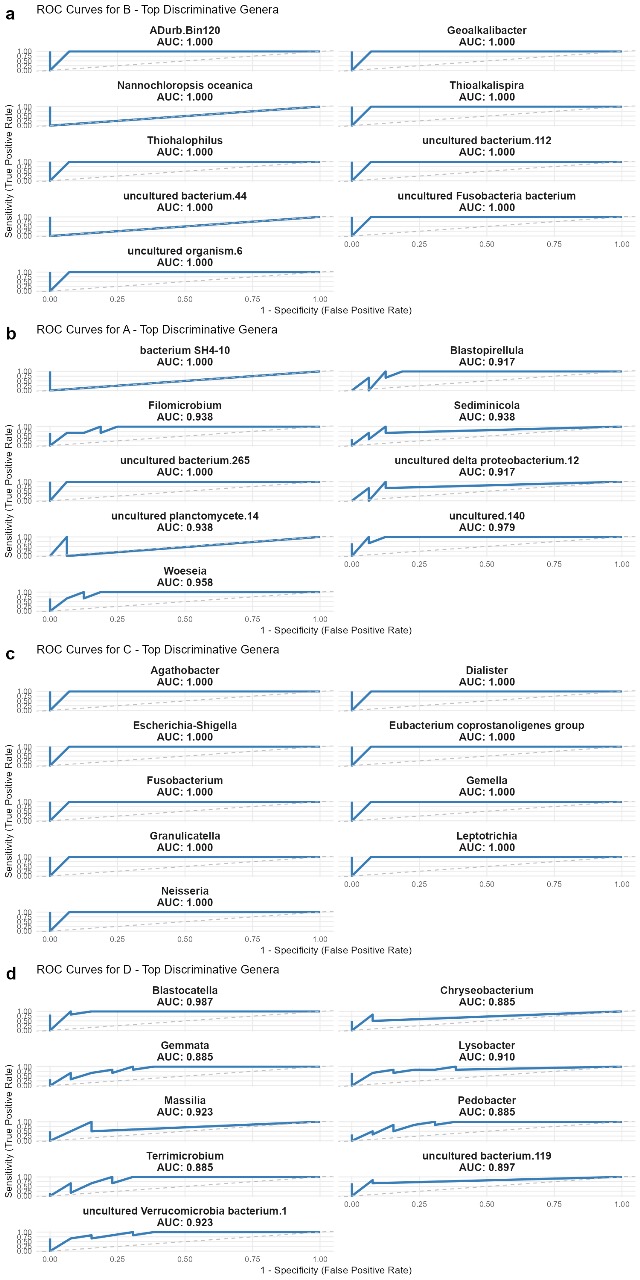


**Figure S4:** A multi-panel plot displays ROC (Receiver Operating Characteristic) curves for the top nine genera most predictive of each group (A, B, C, and D), based on AUC (Area Under the Curve) scores. Each panel represents a genus, annotated with its corresponding AUC value. The curves illustrate the sensitivity versus 1-specificity for each genus in distinguishing its target group from the others. Dashed diagonal lines indicate random classification performance.

**Figure S5: Canonical Correspondence Analysis (CCA) ordination plot of microbial communities.**


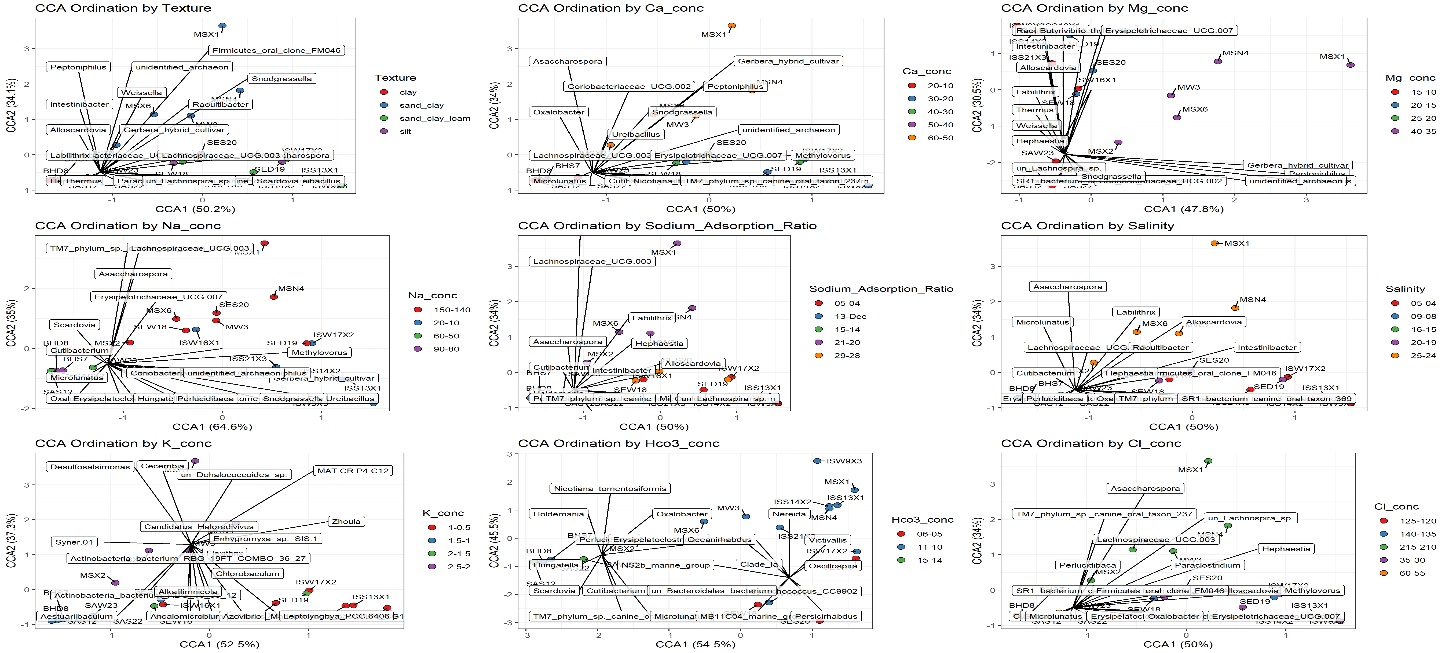


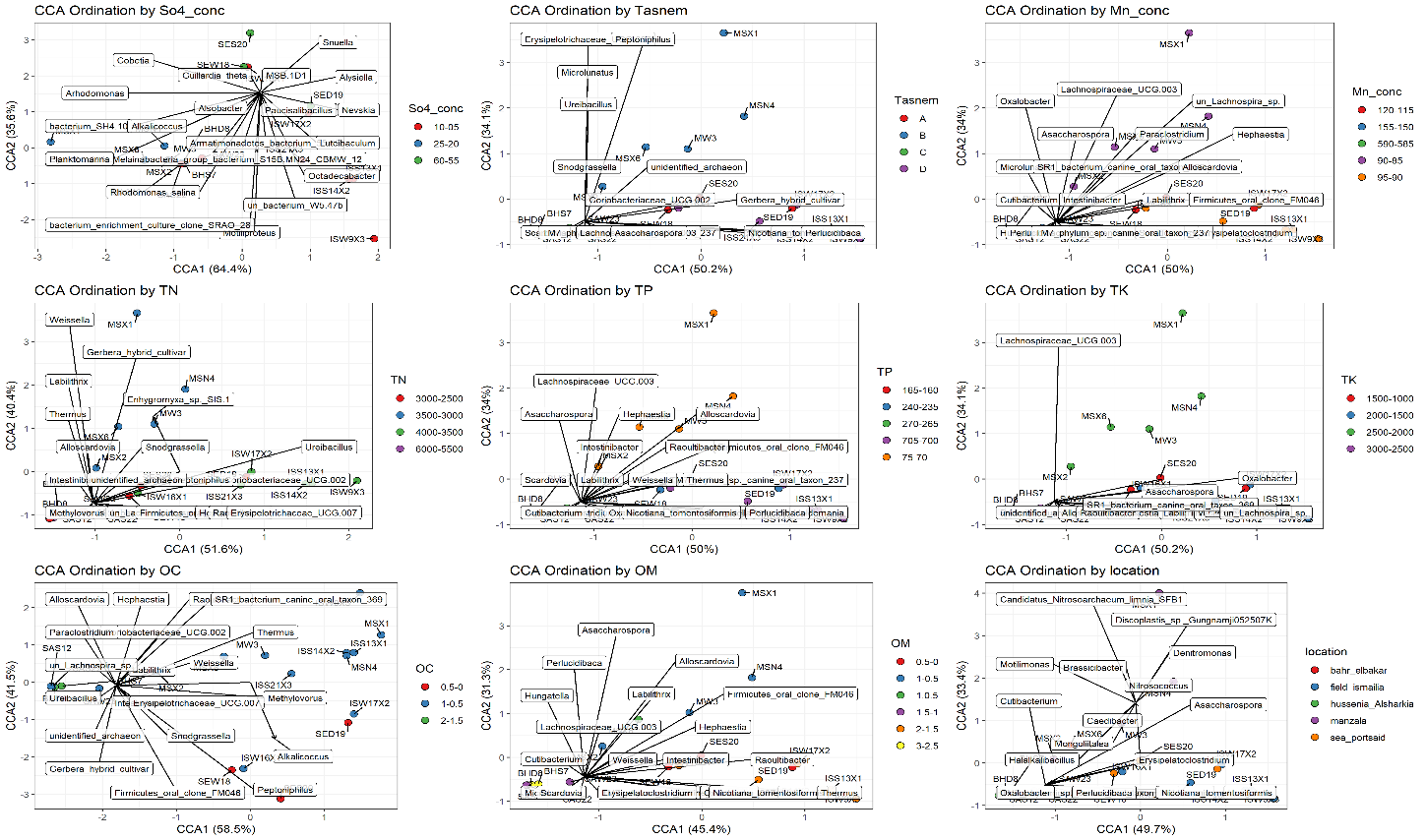


**Figure S5:** Samples are colored and shaped by treatment group: compost amendments, contaminant exposure, or control soils. Red arrows represent the top 20 genera contributing most to community variation, scaled to 70% of their original vector length for clarity. The plot illustrates how microbial composition shifts in response to environmental gradients and treatments.
